# Role of CDK4 as prognostic biomarker in Soft Tissue Sarcoma and synergistic effect of its inhibition in dedifferentiated liposarcoma sequential treatment

**DOI:** 10.1186/s40164-024-00540-4

**Published:** 2024-08-05

**Authors:** Silvia Vanni, Giacomo Miserocchi, Graziana Gallo, Valentina Fausti, Sofia Gabellone, Chiara Liverani, Chiara Spadazzi, Claudia Cocchi, Chiara Calabrese, Giovanni De Luca, Massimo Bassi, Manlio Gessaroli, Nicola Tomasetti, Angelo Campobassi, Federica Pieri, Giorgio Ercolani, Davide Cavaliere, Lorena Gurrieri, Nada Riva, Federica Recine, Toni Ibrahim, Laura Mercatali, Robin Jones, Alessandro De Vita

**Affiliations:** 1 Preclinic and Osteoncology Unit, Biosciences Laboratory, IRCCS Istituto Romagnolo Per Lo Studio Dei Tumori (IRST) “Dino Amadori”, Meldola, FC Italy; 2 Pathology Unit, Bufalini Hospital, Cesena, Italy; 3 Clinical and Experimental Oncology, Immunotherapy, Rare Cancers and Biological Resource Center, IRCCS Istituto Romagnolo Per Lo Studio Dei Tumori (IRST) “Dino Amadori”, Meldola, FC Italy; 4 Maxillofacial Surgery Unit, Bufalini Hospital, Cesena, Italy; 5 Pathology Unit, Morgagni-Pierantoni Hospital, Forlì, Italy; 6 General and Oncologic Surgery, Morgagni-Pierantoni Hospital, Forlì, Italy; 7 Medical Oncology Unit, Azienda Ospedaliera “San Giovanni Addolorata”, Roma, Italy; 8 Osteoncology, Bone and Soft Tissue Sarcomas and Innovative Therapies Unit, IRCCS Istituto Ortopedico Rizzoli, Bologna, 40136 Italy; 9 Sarcoma Unit, The Royal Marsden NHS Foundation Trust, London, UK

## Abstract

Soft tissue sarcomas represent an heterogeneous group of rare mesenchymal tumors comprising 1% of all solid malignancies. Among them, liposarcoma is one of the most common histotypes with atypical lipomatous tumor/well differentiated liposarcoma and dedifferentiated liposarcoma (ALT/WDLPS and DDLPS) as the major sub-entities. The unavailability of predictive, prognostic and druggable biomarkers makes the management of these lesions challenging. In recent years CDK4 and its inhibitors have emerged as potential agents for these lesions especially for ALT/WDLPS and DDLPS but the results are not conclusive and need to be elucidated. This study involved 21 ALT/WDLPS and DDLPS patients. Histological analyses of MDM2 and CDK4 were carried out. Moreover, a DDLPS patient-derived cancer model was established in vitro and in vivo assessing the efficacy of palbociclib in combination and sequential treatment. Finally, *in silico* analyses on CDK4 expression were carried out. The results showed a higher expression of CDK4 and MDM2 in DDLPS compared to ALT/WDLPS. Moreover, no correlation between MDM2 expression and CDK4 was observed. Next, in vitro analysis of CDK4 inhibitor palbociclib showed an antagonistic effect when combined to other chemotherapeutics, while it exhibited a significant synergy when administered in sequential schedule with lenvatinib. Next, in vivo analysis on DDLPS xenotransplanted embryos assessing the efficacy and safety profile of the in vitro tested schedules confirmed the observed data. This proof-of-concept study sheds light on the natural history of ALT/WDLPS and DDLPS and provides the rationale for the clinical applicability of sequential treatment with palbociclib in the management of DDLPS.

**Supplementary Information:**

The online version contains supplementary material available at 10.1186/s40164-024-00540-4.

## To the editor

Among Soft Tissue Sarcoma (STS), representing the 1% of all solid tumors, liposarcomas (LPS) are the most common histotype, which are sub-grouped into four entities including atypical lipomatous tumor (ALT)/well differentiated liposarcoma (WDLPS) and dedifferentiated liposarcoma (DDLPS) [1]. As for many other STS, the unavailability of prognostic and predictive biomarkers represents an urgent clinical need to be solved in order to guide physicians in the patient’s management. Thus, the identification of promising prognostic, predictive and potentially druggable biomarkers could pave the way for innovative strategies in the landscape of sarcoma management. In this regard, in recent years research focusing on the role of cyclin dependent kinase family has emerged in sarcoma [2]. Indeed, from a molecular point of view, STSs frequently harbor the amplification of the 12q13-15 chromosome region, which encodes for different oncogenes including MDM2 and CDK4 [3].

Previous works evaluated the MDM2 and CDK4 expression which resulted in a strong correlation between their amplification and gene status [4]. Their helpful role to differentiate ALT/WDLPS from benign adipose tumors and to distinguish DDLPS from poorly differentiated sarcomas has been underlined. Moreover, the high-CDK4 expression group showed significantly poorer progression free survival (PFS) and disease specific survival than the low-CDK4 expression group [5].

The above results have brought attention to the impairment of CDK4 activity as a potential approach for sarcoma therapy, especially in DDLPS. Thus, a variety of preclinical and clinical studies has been carried out using selective CDK inhibitors [6, 7]. A phase II trial evaluating the activity of CDK4 inhibitor palbociclib in ALT/WDLPS and DDLPS showed that palbociclib administration in advanced disease was associated with a favorable PFS and occasional tumor response [8]. A recent study has highlighted the promising role of adjuvant palbociclib treatment in delaying recurrence in completely resected retroperitoneal LPS [9]. Taking in consideration the above-mentioned data, in this study we aimed to deepen the potential role of CDK4 biomarker for the management of DDLPS providing the rationale for its clinical use.

**Fig. 1:**
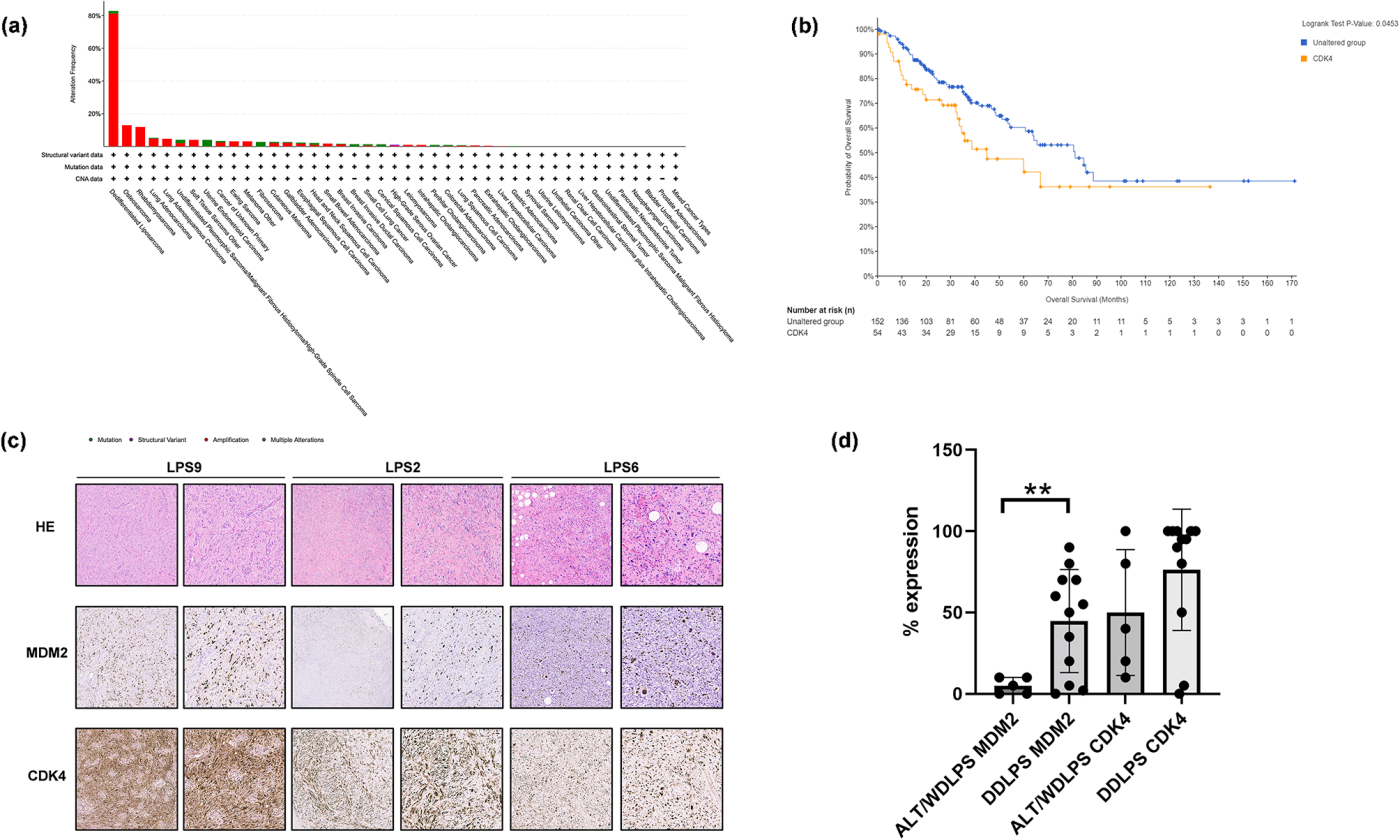
CDK4 expression in STS. (**A**) CDK4 alteration frequency among solid tumors. Red: amplification; Green: Mutation; Grey: Multiple alterations; Blue: Deep deletion. Tumor type groups were included with *n* > 30 cases; (**B**) Overall survival correlation with CDK4 alteration in 206 Soft Tissue Sarcoma; (**C**) Representative images of hematoxylin and eosin (HE) and IHC staining. Upper row: HE of three LPS patients’ surgically resected tumor specimens (10× and 20X magnification). Middle and bottom rows: IHC of MDM2 and CDK4 in LPS patients’ surgically resected tumor specimens (10× and 20X magnification); (**D**) Scatter Plot showing the percentage of expression of MDM2 and CDK4 in ALT/WDLPS and DDLPS, with standard deviation. Significant difference was accepted for *p* < 0.001 (**)

*In silico* analysis of CDK4 alterations among solid tumors underlined its higher mutation frequency in sarcoma compared to all the other malignancies. In particular, among all mutation types, amplification is the most frequent in dedifferentiated liposarcoma (Fig. 1A and Supplementary Fig. 1). Moreover, as shown in Supplementary Fig. 2, patients with CKD4 amplifications also show higher CKD4 mRNA expression. Thus, alteration of CDK4 emerged as a negative prognostic factor for OS in the same case series (Fig. 1B and Supplementary Fig. 3). Therefore, we could infer that higher CDK4 expression is correlated with worse outcome.

Moreover, we focused on the possible correlation between CDK4 and MDM2.

Immunohistochemical analysis on our patient’s tissues case series showed a higher expression of CDK4 and MDM2 in DDLPS compared to ALT/WDLPS (Fig. 1C, D, Supplementary Fig. 4 and Supplementary Table 1). Moreover, although CDK4 and MDM2 are codified by the same genomic region in our case series their expression was not always consistent. Indeed, there are cases with low expression of CDK4 and concomitant high expression of MDM2. Thus, this data suggested that even cases with low MDM2 expression may express CDK4, therefore we could hypothesize that these patients may still benefit from a CDK4 inhibition treatment. Therefore, we aimed to investigate the potential role of CDK4 as a promising biomarker for sarcoma including DDLPS.

**Fig. 2:**
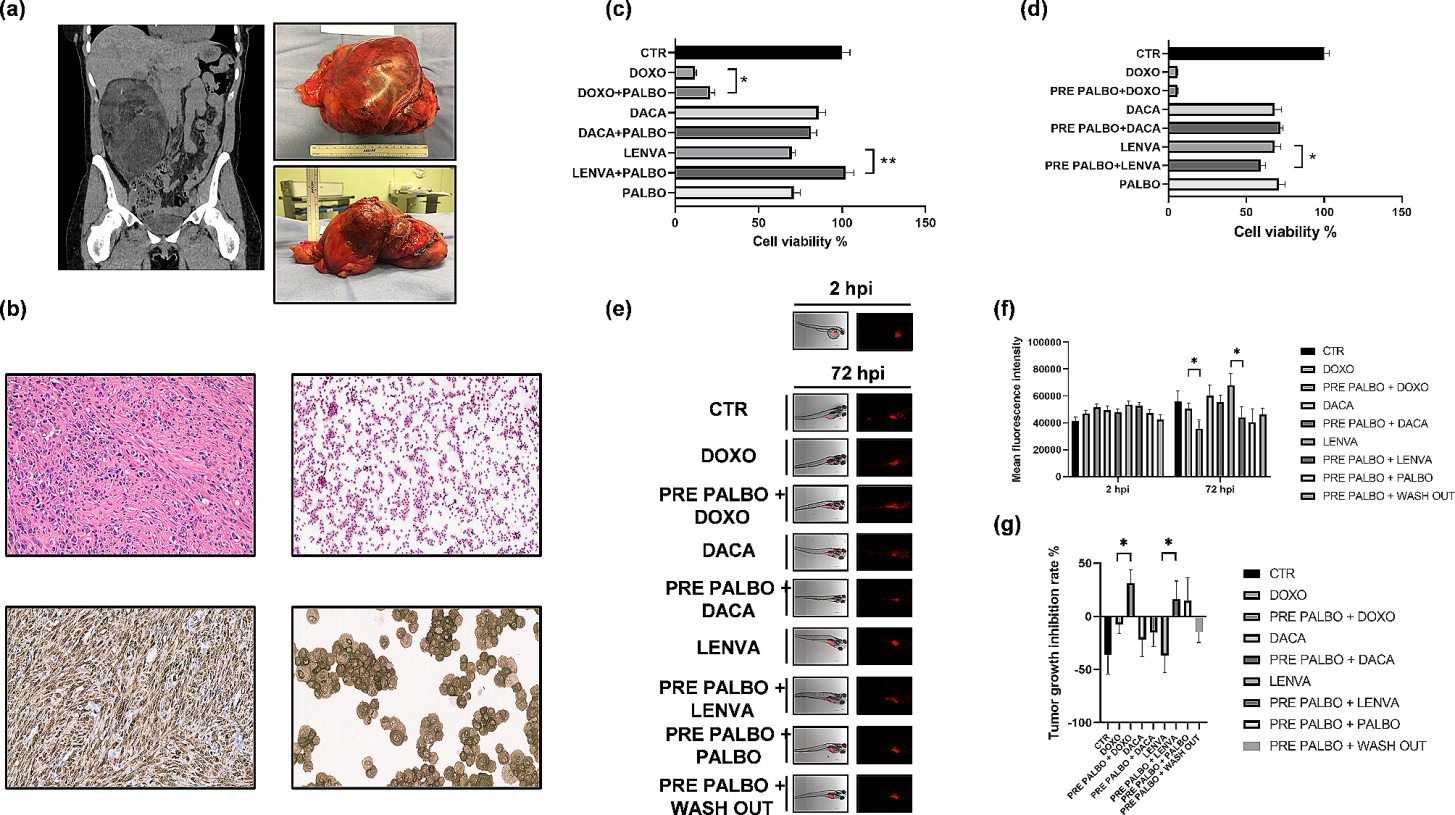
Pharmacological analysis of palbociclib activity in DDLPS patient-derived cells. (**A**) Representative images of DDLPS patient’s frontal CT-scan and resected DDLPS tumor mass and related processed specimen. (**B**) HE and CDK4 IHC staining of patient tumor tissue and patient-derived primary tumor cells. The morphology of the primary culture analyzed through HE staining (upper right) recapitulates the one observed in the patient tumor (upper left) with markedly atypical cells. Moreover, CDK4 expression resulted positive in both the patient tumor and primary culture (lower right and left) further corroborating the establishment of DDLPS patient-derived primary culture model. (**C**) Pharmacological analysis of palbociclib in vitro activity in combination and (**D**) in sequential treatment with chemotherapy in DDLPS patient-derived cells. (*n* = 8 for each condition) (**E**) Representative fluorescence microscopy images of zebrafish embryos xenotransplanted with DDLPS patient-derived primary cells. Representative images of 2 hpi xenotransplanted embryos and images of embryos untreated (CTR) and exposed to tested drugs at 72 hpi, scale bar 1000 μm. (**F**) Mean fluorescence signal of DDLPS xenotransplanted embryos, arbitrary units (n average = 15 for each condition). (**G**) Tumor-growth inhibition rate between tested drugs. Significant differences between treatments were accepted for *p* < 0.05 (*), (n average = 15 for each condition)

Next, we took advantage of patient derived models to assess the role of palbociclib in monoregimen or in combination with first line (doxorubicin, DOXO), second line (dacarbazine, DACA) and off-label (lenvatinib, LENVA) DDLPS treatments. A chemobiogram analysis was carried out combining the use of DDLPS primary cells with in vitro and in vivo models. This approach allows a better recapitulation of human tumor features with respect to common immortalized cell lines. The establishment of a patient-derived primary culture of DDLPS was successfully achieved as confirmed by H&E and CDK4 immunohistochemical staining (Fig. 2A, B). No synergism was observed with the combinations of selected drugs and palbociclib (PALBO) (Fig. 2C). Therefore, we decided to study if palbociclib pre-treatment could increase the synergism of the drug combinations. The pre-administration of palbociclib showed significant reduction in cell viability when used in sequence with lenvatinib (Fig. 2D and Supplementary Fig. 5). Finally, we performed xenografts of DDLPS primary cells in zebrafish embryos to confirm the efficacy of the sequential treatments. In recent years, this kind of approach has been widely used as support for clinical trials, due to its reliability demonstrated in translational studies [10]. In vitro results were partially confirmed by in vivo analysis. Indeed, the results showed a significant reduction in tumor growth with palbociclib-based sequential therapy followed by doxorubicin and lenvatinib (Fig. 2E–G and Supplementary Table 2).

This proof-of-concept study sheds light on the pivotal role of CDK4 as a biomarker in the landscape of STS, especially DDLPS, in terms of prognosis and therapeutic target. Finally, for the first time this work provides evidence for the rationale of a clinical trial evaluating a sequential schedule of palbociclib with anthracycline or lenvatinib treatments for the management of DDLPS patients.

### Electronic supplementary material

Below is the link to the electronic supplementary material.

Supplementary Material 1

Supplementary Material 2

Supplementary Material 3

Supplementary Material 4

Supplementary Material 5

Supplementary Material 6

Supplementary Material 7

Supplementary Material 8

## Data Availability

The datasets generated and/or analysed during the current study are available from the corresponding author on reasonable request.

## Abbreviations

ALT: Atypical lipomatous tumor
CDK4: Cyclin-dependent kinase 4
DDLPS: Dedifferentiated liposarcoma
LPS: Liposarcoma
MDM2: Mouse double minute 2 homolog
PFS: Progression free survival
STS: Soft Tissue Sarcoma
WDLPS: Well differentiated liposarcoma
