# Supplementary material for: Role of CDK4 as prognostic biomarker in Soft Tissue Sarcoma and synergistic effect of its inhibition in dedifferentiated liposarcoma sequential treatment
Source: Exp Hematol Oncol. 2024 Aug 5;13:74. doi: 10.1186/s40164-024-00540-4 (PMC11299298; doi:10.1186/s40164-024-00540-4)
Supplement: Supplementary file 1 — Supplementary Material 1 [file 40164_2024_540_MOESM1_ESM.docx]

**Supplementary material**

1. **Supplementary Materials and Methods**

1.1. In silico analysis

Public dataset repositories were interrogated. In particular cBioPortal for Cancer Genomics (https://www.cbioportal.org/, accessed on 13 June 2024) was used for CDK4 alteration frequency expression in silico analysis among 10.953 patients affected by solid tumors (cancer types detailed). Finally, the expression of CDK4 was correlated to the overall survival and disease free survival of a case series of 206 soft tissue sarcoma.

1.2. Ethical approval and case series study

All human samples were anonymized. The study involved 20 adult patients, 14 male and 6 female patients, with a median age of 69 years, affected by liposarcoma (n= 7 ALT/WDLPS and n= 13 DDLPS) (Supplementary Table 1). IRST-Area Vasta Romagna Ethics Committee approved the study protocol, approval no. 4751, 31 July 2015. Good Clinical Practice standard operating procedures and 1975 Helsinki declaration were applied in the study. Informed consent for participation in the research study was obtained from each patient. Enrolment of patients started in July 2011 and ended in March 2022.

1.3. Histological Analyses

Cytomorphological evaluation and tumor architecture analysis were investigated through hematoxylin and eosin (H&E) staining. In brief, resected tumor specimens were paraffin embedded and sectioned into 5-µm-thick slices using a microtome. For immunohistochemical analysis (IHC), tumor tissues were de-paraffinized for 1 h using xylene and then rehydrated and incubated with antigen retrieval solution in a water bath at 98.5 ◦C for 30 min. Next, the sections were incubated with a 3% hydrogen peroxide solution for 10 min and washed twice with demineralized water. After washes, slices were blocked with a 3% bovine serum albumin solution in PBS for 20 min and then incubated at room temperature for 1 h with antibodies: MDM2 (Monoclonal Antibody IF2, Thermo Fisher Scientific) and CDK4 (Rabbit Recombinant Monoclonal Antibody EPR4513-32-7, Abcam). Streptavidin-biotin-peroxidase complex (ABC) method was used for revealing the staining and hematoxylin was used for counterstaining the cell nuclei. Staining positivity was considered in the presence of brown nuclear immunostaining.

1.4. Establishment of a patient-derived DDLPS model

The patients signed informed written consent before undergoing surgical treatment. An experienced pathologist analyzed the tissue samples before the transport to the Biosciences Laboratory of our institute (IRST IRCCS). Tumor tissues were minced into pieces of 0.1 to 1 mm3 using surgical scalpels. A solution of PBS and 2 mg/mL collagenase type I (Millipore Corporation) 1:1 was used for the enzymatic digestion. The fragment suspension was incubated for 15’ at 37 °C and then at room temperature for a further 15 min. The obtained cell suspension was filtered in order to eliminate the cell's debris and digested tumor matrix. Isolated tumor cells were cultured in standard monolayer cultures with a cell density of 80,000 per cm^2^. DDLPS primary cells were maintained in DMEM supplemented with 10% fetal bovine serum (Invitrogen), 1% penicillin/streptomycin and 1% glutamine at 37 ◦C in a 5% CO_2_ atmosphere. Media refresh was performed twice a week. All experiments were performed using low-passage and actively proliferating primary cells. Hematoxylin and eosin (H&E) staining of isolated DDLPS patient-derived primary cells was performed in order to evaluate their histopathological features according to the manufacturer’s instructions. Briefly, for standard monolayer cultures 100,000 cells were cytospun onto glass slides and fixed. H&E stained slides were analyzed and images were captured with an optical Zeiss Axioskop microscope. For IHC analysis of CDK4 the protocol used was the same used for tumor tissue and reported in the histological analyses section.

1.5. In vitro drug testing

Patient-derived primary cultures were exposed to different drugs and their efficacy was quantified through a 3-(4,5-dimethylthiazol-2-yl)-2,5-diphenyltetrazolium bromide (MTT) reduction assay. Briefly, liposarcoma primary cells were seeded in 96-well plates at a density of 80,000 cells/cm^2^ and exposed to drugs for 72 h. Drugs regimen were selected according to the plasma peak of each drug obtained from pharmacokinetic clinical data in patients affected by solid tumors: Doxorubicin (DOXO) 4 µg/mL (Accord Healthcare Ltd., Milan, Italy), Dacarbazine (DACA) 8 ug/mL (Medac Pharma, Rome, Italy), Lenvatinib (LENVA) 0.6 µg/mL (Eisai Ltd., Milan, Italy), Palbociclib (PALBO) 97 ng/mL (MedChemExpress LLC, Monmouth Junction, NJ, USA) (Braal et al., 2021). Cell survival percentage was quantified after 72 h of drug exposure. The experiments were performed twice.

1.6. Combination and sequential treatments

Chemobiogram analysis on DDLPS patient-derived primary cultures was assessed through MTT reduction assay as reported in the drug testing section. Primary cells were exposed for 72h to both chemotherapeutic drugs in monoregimen or in combination with PALBO.

For sequential treatments DDLPS patient-derived primary cultures were exposed for 24 h to PALBO and then for 48 h to the selected chemotherapeutic drugs in the combination treatments section. The tested conditions without sequential treatment were refreshed with new media after 24 h.

1.7. Wound healing assay

Wound healing assay was performed according to manufacturer's instructions using Culture-Insert 2 Well in µ-Dish 35 mm (Ibidi, Gräfelfing, Germany). Briefly, 15 x 103 DDLPS patient-derived primary cells were seeded in the culture dishes and after 24 h they were exposed for 10 h to PALBO (pre-treatment) and subsequently for 10 h to chemotherapy including DOXO, DACA and LENVA. Control group media was washed out and replaced with new media. Images were captured with EVOS XL Cell Imaging System (Thermo Fisher Scientific, Waltham, MA, USA) at 0 and 24 h after treatment and the cell migration rate was calculated by determining the wound closure after 24 h treatment exposure and compared to untreated cells. Delta percentage was obtained via the following: (cell free surface area observed at 24h / cell free surface area observed at 0h) x 100

1.8. In vivo drug testing

AB wild type zebrafish embryos were housed according to Kimmel et al.. Briefly, embryos were dechorionated at 48 h post fertilization (hpf) and xenotransplanted into the yolk sack with 300/500 DDLPS primary cells labeled with a red fluorescent dye (CellTracker™ CM-DiI, Invitrogen). Grafted embryos were sorted in nine groups and exposed to 8 drug schedules (20 zebrafish embryos each condition) and used for downstream analysis.

DDLPS xenotransplanted embryos were exposed for 72 h to the assessed chemotherapeutic drugs. For sequential treatments embryos were exposed for 24 h to PALBO and then for 48 h to the assessed chemotherapeutic drugs. Next, embryos were incubated at 32 °C and then imaged for tumor area using a fluorescence stereomicroscope (Nikon SMZ 25 equipped with NIS Elements software).

1.9. *In vivo* drug toxicology study

In order to investigate the in vivo safety of sequential treatments AB wild type zebrafish embryos were sorted in 9 groups and exposed to the investigated chemotherapeutic drugs or the sequential treatment with PALBO following the assessed drugs (20 zebrafish embryos each condition). Next, embryos were incubated at 32 °C and then imaged for severe abnormalities including pericardial edema, swim bladder deficiency and yolk retention.

1.10. Statistical Analysis

Each experiment was conducted with two or three independent replicates. Data are presented as mean ± standard deviation or standard error (n = the number of replicates). Differences between groups were assessed by a two-tailed Student’s t-test and accepted as significant at p < 0.05.
