# Supplementary material for: Role of CDK4 as prognostic biomarker in Soft Tissue Sarcoma and synergistic effect of its inhibition in dedifferentiated liposarcoma sequential treatment
Source: Exp Hematol Oncol. 2024 Aug 5;13:74. doi: 10.1186/s40164-024-00540-4 (PMC11299298; doi:10.1186/s40164-024-00540-4)
Supplement: Supplementary file 2 — Supplementary Material 2 [file 40164_2024_540_MOESM2_ESM.docx]

| Code | Sex | Age | Site | Diagnosis | Primitive | Local recurrence | Metastasis | CDK4 positivity | CDK4 % | MDM2 positivity | MDM2 % | Subsequent metastasis |
| --- | --- | --- | --- | --- | --- | --- | --- | --- | --- | --- | --- | --- |
| LPS1 | M | 77 | spermatic cord | DDLPS | X |  |  | + | 100 | + | 60 |  |
| LPS2 | F | 69 | skin | DDLPS | X |  |  | + | 90 | + | 2 |  |
| LPS3 | M | 63 | submental | DDLPS | X |  | X | + | 95 | + | 55 |  |
| LPS4 | M | 74 | spermatic cord | DDLPS | X |  |  | + | 100 | + | 50 |  |
| LPS5 | F | 49 | abdomen | DDLPS | X |  |  | + | 95 | + | 70 |  |
| LPS6 | M | 69 | retroperitoneal | DDLPS | X |  |  | + | 50 | + | 70 | Lung |
| LPS7 | M | 84 | retroperitoneal | DDLPS | X |  | X | + | N/A | + | 80 |  |
| LPS8 | M | 82 | spermatic cord | DDLPS | X |  |  | + | 100 | + | 35 |  |
| LPS9 | M | 85 | spermatic cord | DDLPS | X |  |  | + | 100 | + | 20 |  |
| LPS10 | M | 72 | retroperitoneal | DDLPS | N/A |  |  | + | 5 | + | 5 |  |
| LPS11 | M | 62 | spermatic cord | DDLPS | N/A |  |  | + | 80 | - | 0 |  |
| LPS12 | F | 75 | renal lodge | DDLPS | X |  |  |  |  |  |  | N/A |
| LPS13 | M | 82 | cheek | DDLPS and ALT/WDLPS |  | X |  | + | 100 | + | 90 |  |
| LPS14 | M | 54 | abdomen | ALT/WDLPS | X |  |  | + | 80 | + | 10 |  |
| LPS15 | M | 82 | thigh | ALT/WDLPS | X |  |  | + | 40 | - | 0 |  |
| LPS16 | F | 62 | retroperitoneal | ALT/WDLPS | X |  |  | + | 10 | - | 0 | kidney |
| LPS17 | F | 64 | thigh | ALT/WDLPS | X |  |  | + | 20 | + | 5 | low arm |
| LPS18 | M | 55 | bib | ALT/WDLPS | X |  |  |  |  |  |  | N/A |
| LPS19 | F | 50 | small basin | ALT/WDLPS | N/A |  |  |  |  |  |  | N/A |
| LPS20 | M | 79 | hemiscrote | ALT/WDLPS | X |  |  | + | 100 | + | 10 |  |
