# Supplementary figures and images for: Role of CDK4 as prognostic biomarker in Soft Tissue Sarcoma and synergistic effect of its inhibition in dedifferentiated liposarcoma sequential treatment
Source: Exp Hematol Oncol. 2024 Aug 5;13:74. doi: 10.1186/s40164-024-00540-4 (PMC11299298; doi:10.1186/s40164-024-00540-4)

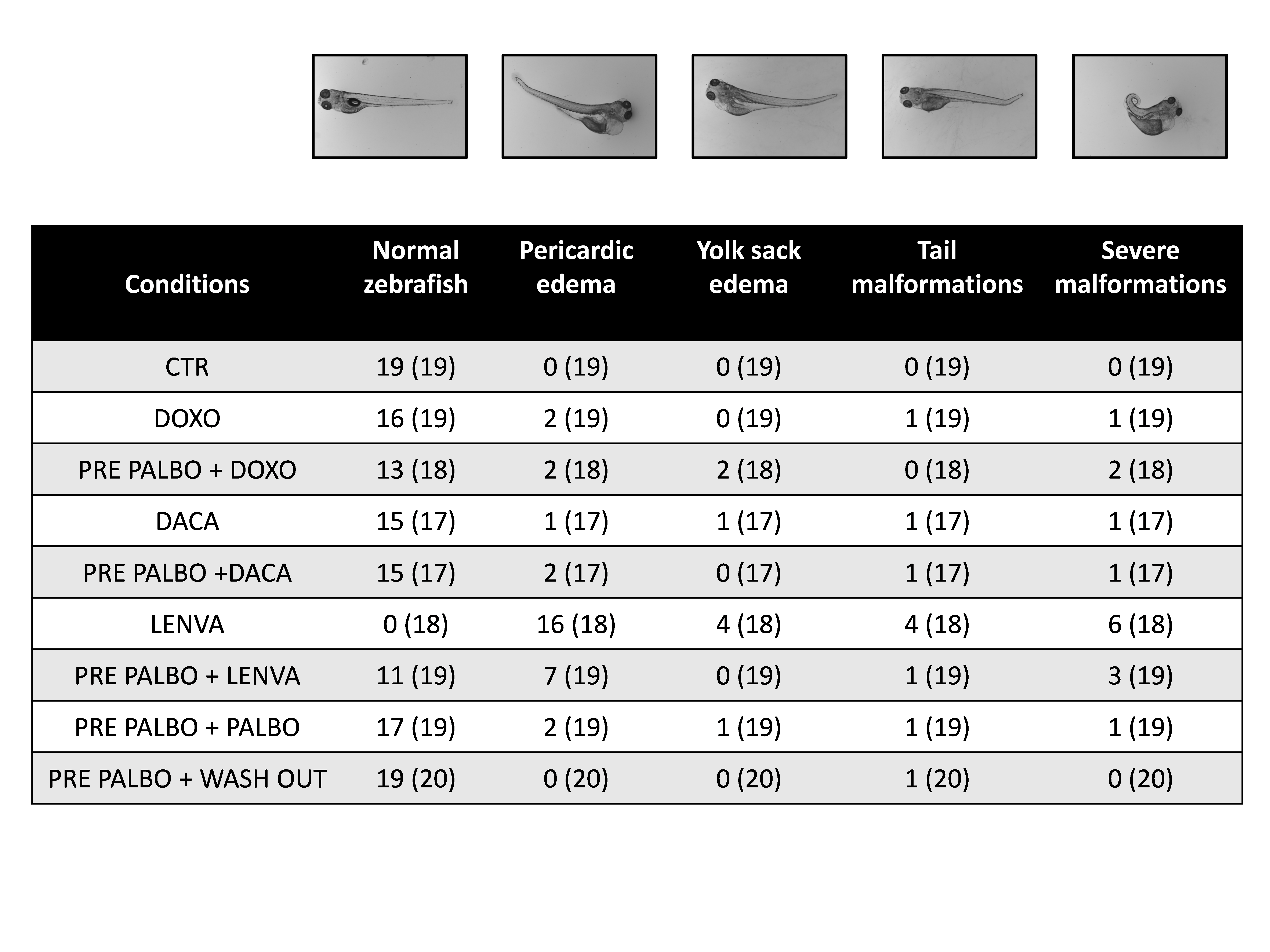

Supplement: Supplementary file 3 — Supplementary Material 3 [file 40164_2024_540_MOESM3_ESM.png]

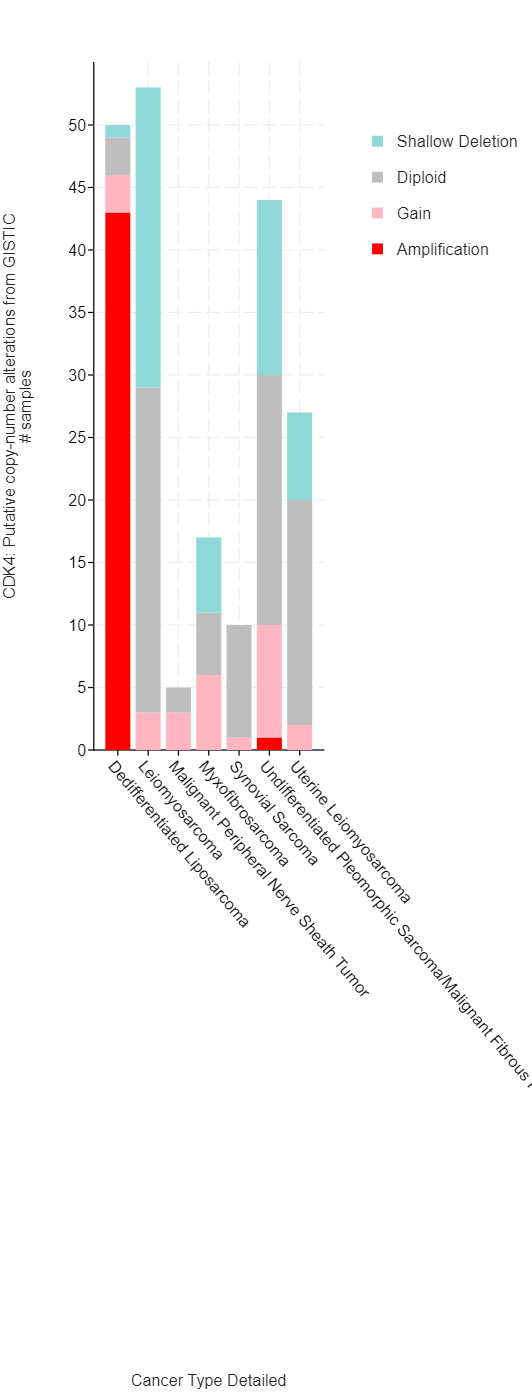

Supplement: Supplementary file 4 — Supplementary Material 4 [file 40164_2024_540_MOESM4_ESM.png]

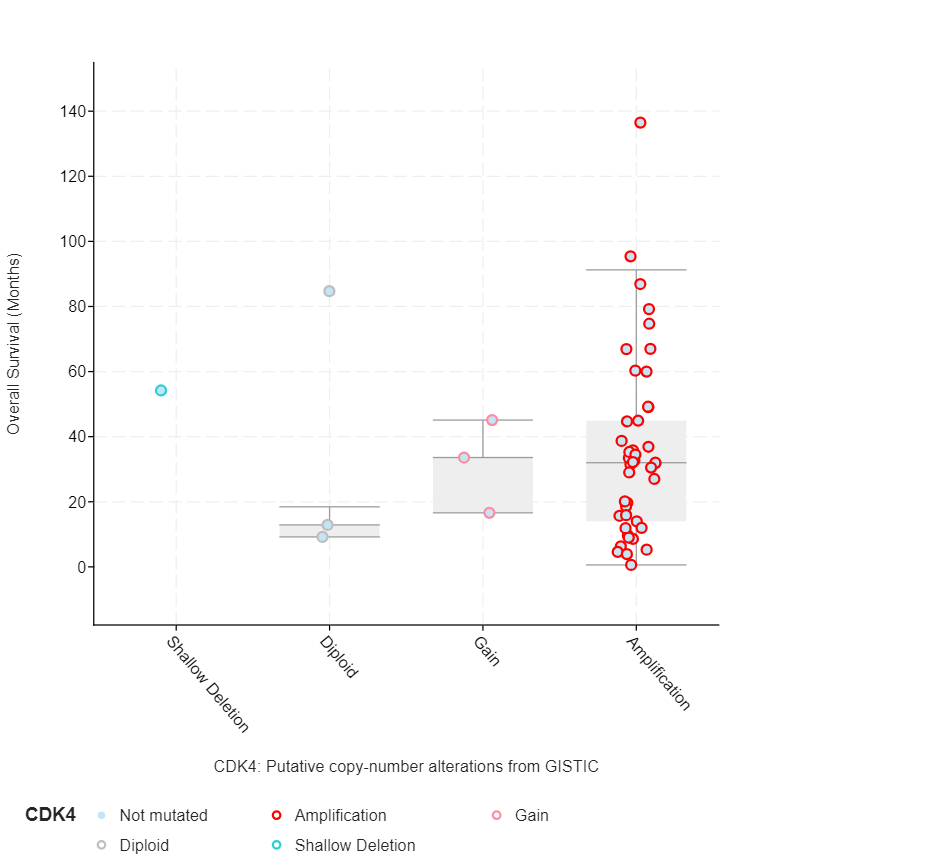

Supplement: Supplementary file 5 — Supplementary Material 5 [file 40164_2024_540_MOESM5_ESM.png]

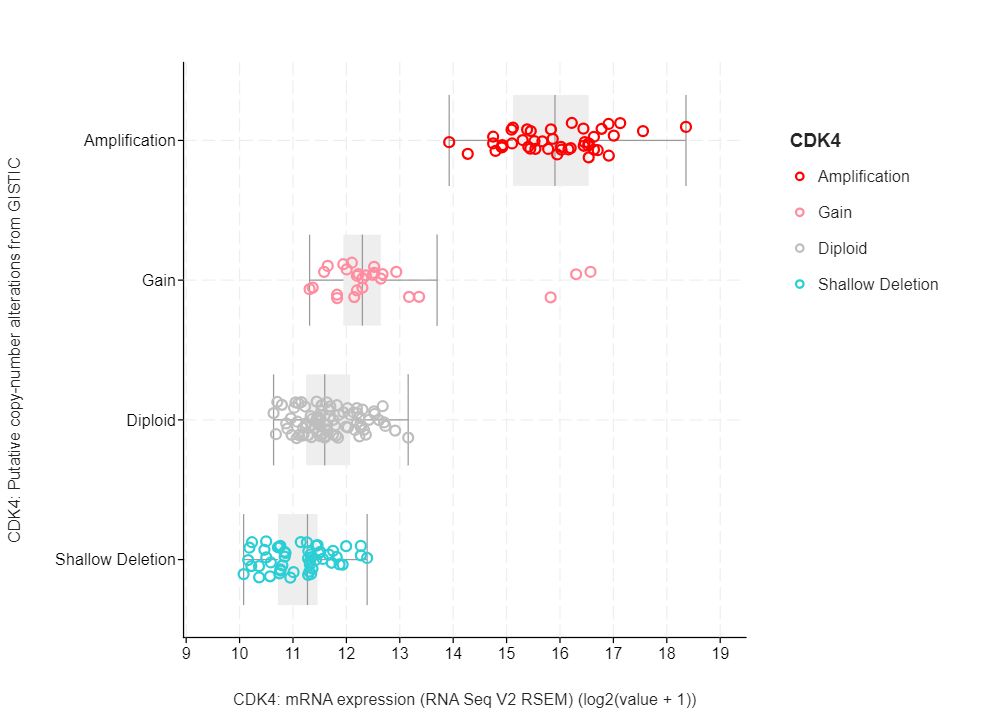

Supplement: Supplementary file 6 — Supplementary Material 6 [file 40164_2024_540_MOESM6_ESM.png]

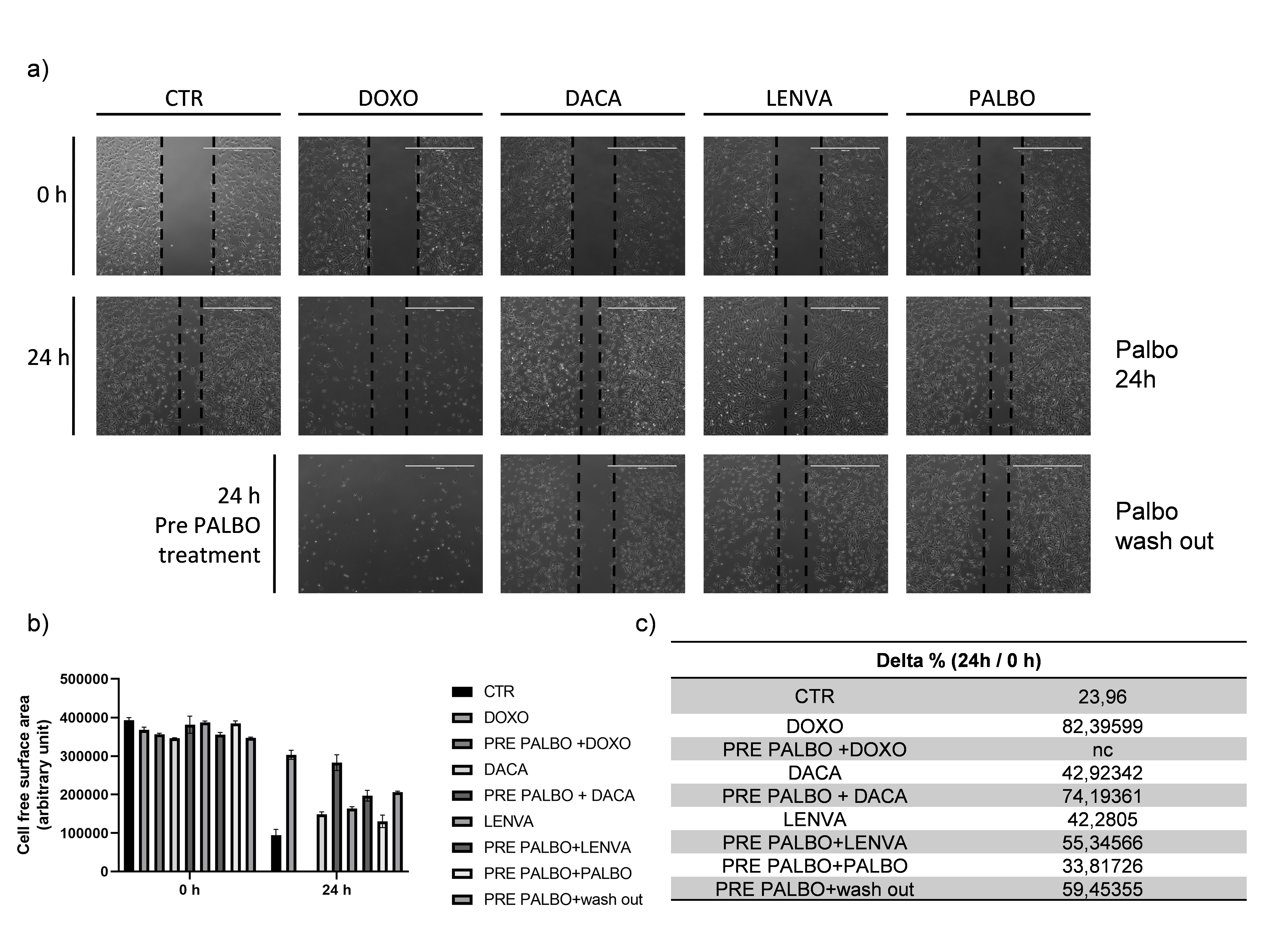

Supplement: Supplementary file 7 — Supplementary Material 7 [file 40164_2024_540_MOESM7_ESM.png]

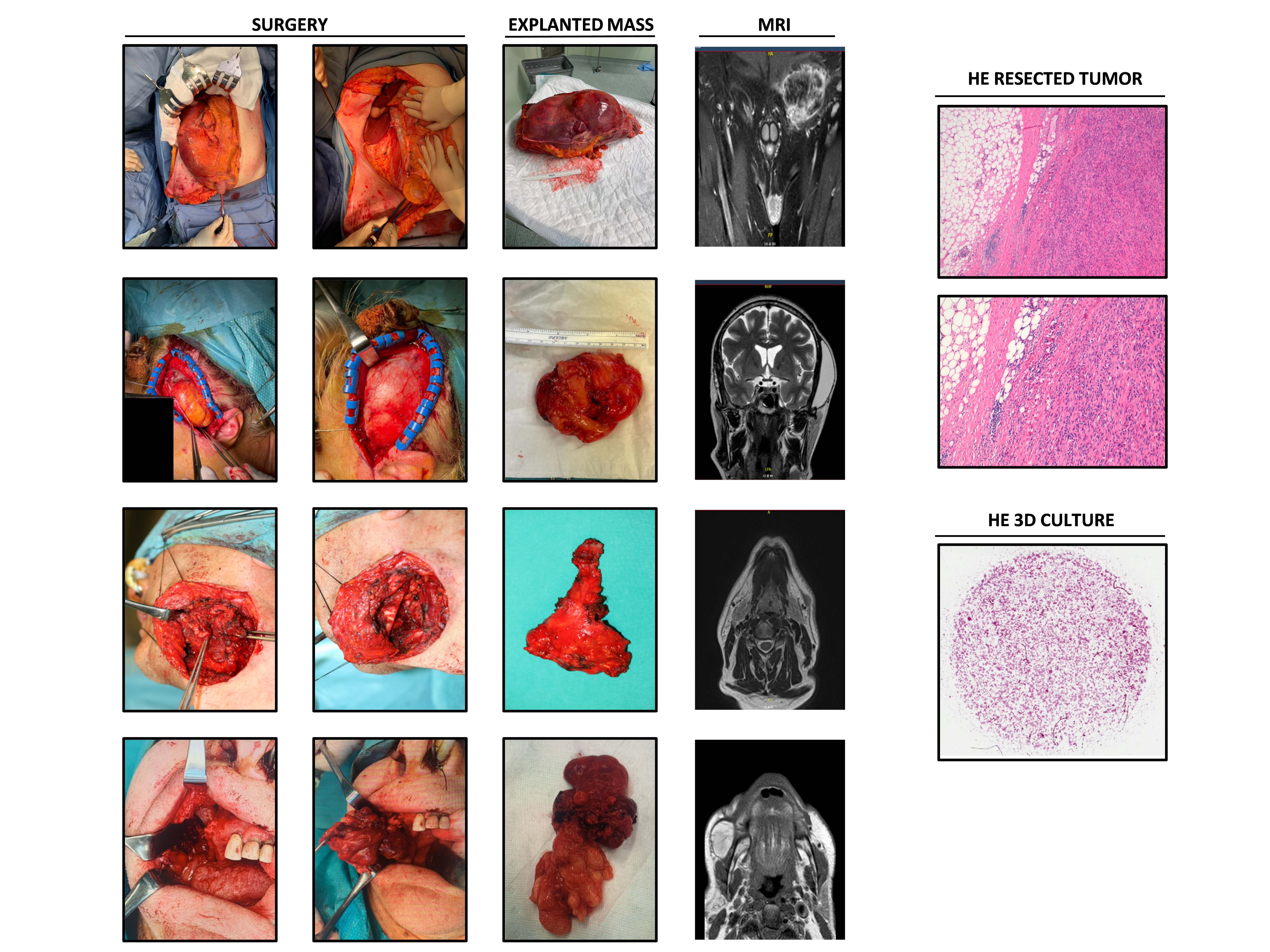

Supplement: Supplementary file 8 — Supplementary Material 8 [file 40164_2024_540_MOESM8_ESM.png]
